# Optimized strategy for schistosomiasis elimination: results from marginal benefit modeling

**DOI:** 10.1186/s13071-023-06001-x

**Published:** 2023-11-15

**Authors:** Qin Li, Jin-Xin Zheng, Tie-Wu Jia, Xin-Yu Feng, Chao Lv, Li-Juan Zhang, Guo-Jing Yang, Jing Xu, Xiao-Nong Zhou

**Affiliations:** 1 National Institute of Parasitic Diseases, Chinese Center for Disease Control and Prevention (Chinese Center for Tropical Diseases Research), National Health Commission Key Laboratory of Parasite and Vector Biology, WHO Collaborating Centre for Tropical Diseases, National Center for International Research on Tropical Diseases, Shanghai, 200025 China; 2 Ruijin Hospital Affiliated to The Shanghai Jiao Tong University Medical School, Shanghai, 200025 China; 3 School of Tropical Medicine, Hainan Medical University, Haikou, 571199 China; 4 School of Global Health, Chinese Center for Tropical Diseases Research and Shanghai Jiao Tong University School of Medicine, One Health Center, Shanghai Jiao Tong University and The Edinburgh University, Shanghai, 200025 China

**Keywords:** Schistosomiasis elimination, Marginal benefit analysis, Machine learning analysis, Modeling, Integrated control strategy, Cost-effectiveness, Optimization

## Abstract

**Background:**

Poverty contributes to the transmission of schistosomiasis via multiple pathways, with the insufficiency of appropriate interventions being a crucial factor. The aim of this article is to provide more economical and feasible intervention measures for endemic areas with varying levels of poverty.

**Methods:**

We collected and analyzed the prevalence patterns along with the cost of control measures in 11 counties over the last 20 years in China. Seven machine learning models, including XGBoost, support vector machine, generalized linear model, regression tree, random forest, gradient boosting machine and neural network, were used for developing model and calculate marginal benefits.

**Results:**

The XGBoost model had the highest prediction accuracy with an *R*^2^ of 0.7308. Results showed that risk surveillance, snail control with molluscicides and treatment were the most effective interventions in controlling schistosomiasis prevalence. The best combination of interventions was interlacing seven interventions, including risk surveillance, treatment, toilet construction, health education, snail control with molluscicides, cattle slaughter and animal chemotherapy. The marginal benefit of risk surveillance is the most effective intervention among nine interventions, which was influenced by the prevalence of schistosomiasis and cost.

**Conclusions:**

In the elimination phase of the national schistosomiasis program, emphasizing risk surveillance holds significant importance in terms of cost-saving.

**Graphical Abstract:**

**Figure:**
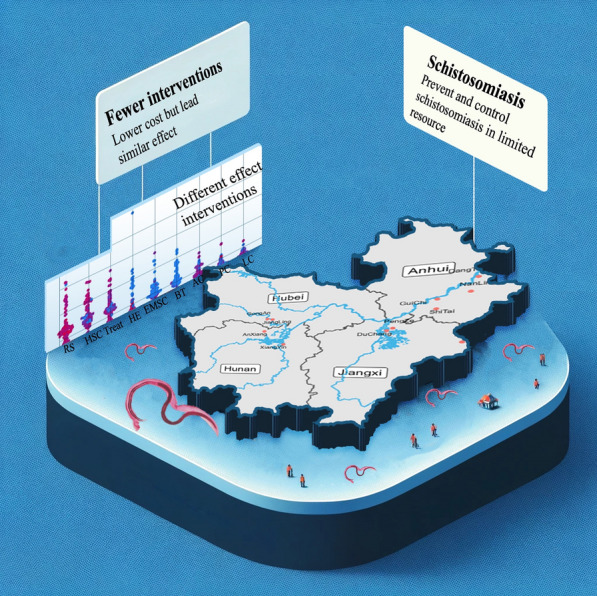

**Supplementary Information:**

The online version contains supplementary material available at 10.1186/s13071-023-06001-x.

## Background

Schistosomiasis japonica is a zoonotic disease caused by infection with the parasitic *Schistosoma japonicum*, which has resulted in millions of deaths and significant social and economic impacts in Southeast Asia, including China, Indonesia, the Philippines and other countries [1]. In China, an integrated intervention strategy with multi-interventions, performed with the National Schistosomiasis Control Program initiated since 2004 [2, 3], has significantly reduced the disease prevalence [4, 5]. According to the survey data from 2021, the total number of infected individuals in China has decreased to 29,041, with approximately 75.17% of endemic counties having achieved disease elimination and only 2.66% of counties still in the transmission control stage [6]. China’s efforts in schistosomiasis control entered the final elimination stage in 2014 [7].

In the elimination stage, as the schistosomiasis prevalence situation evolves, the originally formulated control strategies with the combination of multi-interventions may require adjustments. This is because different regions may experience varying epidemic conditions, with some areas having achieved significant progress with control efforts, while others still face considerable challenges. Consequently, it is crucial to allocate resources based on the benefits of control measures in each region, which will contribute to a more efficient advancement of schistosomiasis elimination [8].

Marginal benefit analysis serves as a tool for studying resource allocation and has been widely applied in various fields [9, 10]. Marginal benefit analysis supported by machine learning (ML) is able to provide information to both resource allocation and better adjust control interventions [11]. ML explores hidden patterns in data through iterative algorithms [12, 13]. Particularly, many tools are available to help us understand the workings of ML, explain their predictions and assess the importance of features and model performance. These interpretability tools have been widely applied in various fields for calculating the benefits of different inputs and optimizing combinations [14, 15]. Therefore, this study can apply this approach to calculate the marginal benefits of different interventions.

This study aims to propose intervention strategies that are better suited to endemic regions with varying geographical characteristics during the elimination stage by calculating the marginal benefits of multi-interventions.

## Methods

### Study sites

In this study, we selected sampling sites from schistosomiasis-endemic counties in the middle and lower reaches of the Yangtze River. These counties were categorized as lake or mountainous endemic based on their terrain and transmission characteristics, with the proportion of each type determined according to the national average.

The inclusion criteria included: (i) meet the criteria for schistosomiasis transmission interruption after 2015; (ii) completion rate > 90% for data availability on schistosomiasis intervention costs and schistosomiasis prevalence from 2002 to 2021; (iii) average annual GDP growth rate in schistosomiasis-endemic counties from 2002 to 2021 was 8.2%, with a standard deviation of 3.5. By excluding counties with significant deviations from the mean, we applied outlier theory for the calculations. Exclusion criteria were (i) not part of a schistosomiasis-endemic area in China; (ii) transmission interruption criteria has not yet met at county level; (iii) percentage of missing data for schistosomiasis prevalence and intervention cost data more than 10%.

### Data collection and processing

This study collected data from 2002 to 2022. Three types of databases were established: (i) an epidemiological database, which includes variables of human infections, animal infections, snail infections, snail habitants, etc.; (ii) a database of interventions cost, which includes cost of risk surveillance, molluscicide for snail control, treatment, population chemotherapy, building toilets, environmental modification for snail control, animal slaughter, health education and livestock chemotherapy; (iii) an economical database, which includes GDP, value added of primary industry, secondary industry, tertiary industry, etc. The variables for epidemical data and economic development status are listed in Table 1, and the intervention cost data are listed in Table 2.

**Table 1:** Sampling counties with epidemiological and economic variables

ID	County name	Epidemy type	Year^a^	GDP growth rate in 2022^b^
1	Dangtu County in Anhui Province	Lake region	2022	13.88
2	Nanling County in Anhui Province	Mountainous region	2019	12.96
3	Shitai County in Anhui Province	Mountainous region	2020	9.18
4	Anxiang County in Hunan Province	Lake region	2019	6.29
5	Xiangyin County in Hunan Province	Lake region	2019	6.38
6	Duchang County in Jiangxi Province	Lake region	2022	14.68
7	Pengze County in Jiangxi Province	Mountainous region	2023 as planed	14.47
8	Yushan County in Jiangxi Province	Mountainous region	2019	14.31
9	Guichi County in Anhui Province	Lake region	2019	11.61
10	Jiangling County in Hubei Province	Lake region	2017	7.91
11	Gongan County in Hubei Province	Lake region	2018	5.93

^a^Year: year of achieving transmission interruption standard

^b^GDP growth rate in 2022 = GDP (2022)−GDP (2002)/GDP (2002); data are collected from China statistical yearbook (county level)

**Table 2:** Specific measures included in each intervention

Intervention	Abbreviation	Context
Risk surveillance	RS	The cost of surveillance in endemic areas, including surveillance human infections, animal infections, snail infections and snail habitats as well as the routine operation of surveillance stations
Molluscicide for snail control	MSC	The cost of using molluscicides to control or eliminate snails, including the procurement of molluscicides, hiring of labor and renting of equipment for the application of molluscicides
Treatment	Treat	Government subsidies provided for the diagnosis, medication and supportive care of schistosomiasis patients
Population chemotherapy	PC	The cost associated with the mass drug administration (MDA) of praziquantel to the population in the endemic area, regardless of whether there are clear symptoms or cases of infection
Building toilets	BT	The cost of renovating and constructing toilets
Environmental modification for snail control	EMSC	The cost of snail elimination through physical and biological control measures (such as snail-suppressive forests, irrigation hardening)
Animal slaughter	AR	The cost of eliminating reservoir hosts (e.g., cattle, sheep, etc.) replaced by machine in endemic areas
Health education	HE	The cost of disseminating knowledge to the public about the pathogen transmission, disease prevention and promoting community engagement and behavior change interventions
Livestock chemotherapy	LC	The cost of administering drug therapy to livestock in endemic areas

We utilized linear interpolation to handle the missing data. Covariance tests were performed to ensure accurate analysis of the data. Before modeling, the costs are discounted to its equivalent value in 2021 at an annual discount rate of 3% using the following formula:

$${\mathrm{C}}_{o}={\mathrm{C}}_{t}/{(1+r)}^{t}$$

In the above equation, r is the annual discount rate, ${C}_{o}$ is the cost at the start of the analysis, and ${C}_{t}$ is the cost at time t.

### Modeling and calculating marginal benefits

**Fig. 1:**
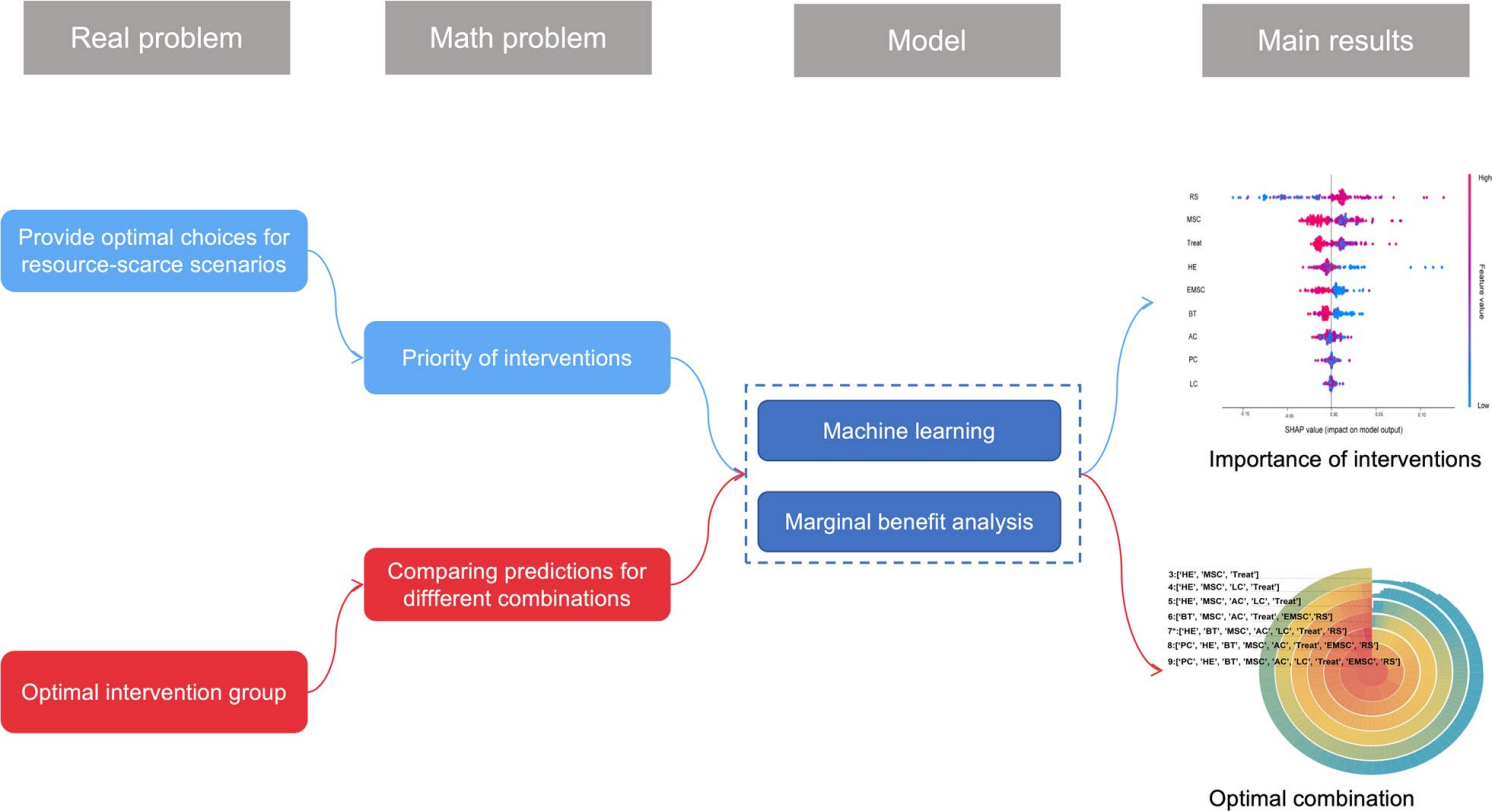
Research methodology diagram. This study originates from two real-world issues, which are transformed into mathematical problems, and models are then constructed based on these problems. The model results are used to propose potential references that offer solutions to the real-world issues

We developed machine learning models following the following four steps (Fig. 1). Prevalence is the ratio of people with schistosomiasis to the total population in the endemic area. After adding 1 to the cost data and taking the logarithm, it was input into the model to calculate the marginal benefit.

First, we developed seven models, including XGBoost, support vector machine, generalized linear model, regression tree, random forest, gradient boosting machine and neural network [13] (Additional file 1). All data were randomly split into a training set and a test set, with a 7:3 ratio [16]. Second, the model evaluation was performed to test the difference or residuals between the predicted and real data. Four evaluation assessments, including R^2^, MSE, RMSE and MAE, were used to assess the model performance (Table 2) [17]. The larger R^2^ represented a higher fitting accuracy of the model, while the smaller MSE, RMSE and MAE represented a higher fitting accuracy of the model. Third, parameter optimization. We used grid search cross-validation to optimize machine learning model parameters [18] (Additional file 2). It exhaustively listed all possible values within the parameter space, evaluated the performance of each parameter combination through cross-validation and identified the optimal hyperparameter. Fourth, this study employed the interpretative functionality of Shapley Additive exPlanations (SHAP) to estimate the marginal effects of various interventions for each endemic county from 2002 to 2021 [19].

### Estimation of the optimal strategies using marginal benefits

Initially, the endemic-prone regions are classified into different endemic regions, namely mountainous and lake regions. Subsequently, interventions for distinct endemic-prone regions are grouped based on the following methodology: a total of nine interventions under study were randomly combined into groups of various sizes. For example, new combinations were formed by randomly selecting three interventions from the complete set, then selecting four interventions separately for new combinations and continuing this process until new combinations included all nine interventions.

The additivity feature of SHAP implies that the contribution of a new combination can be obtained by summing the SHAP values of included interventions [20]. Lastly, the optimal combination was identified through the utilization of metrics including R^2^, MSE, RMSE and MAE. The total cost of each new combination was estimated by summing the average costs of included interventions (Additional files 3, 4).

All data analysis and plotting in this study were performed using R (4.1.0 version) and Python (3.11.4 version) software.

## Results

### Sampled datasets

**Fig. 2:**
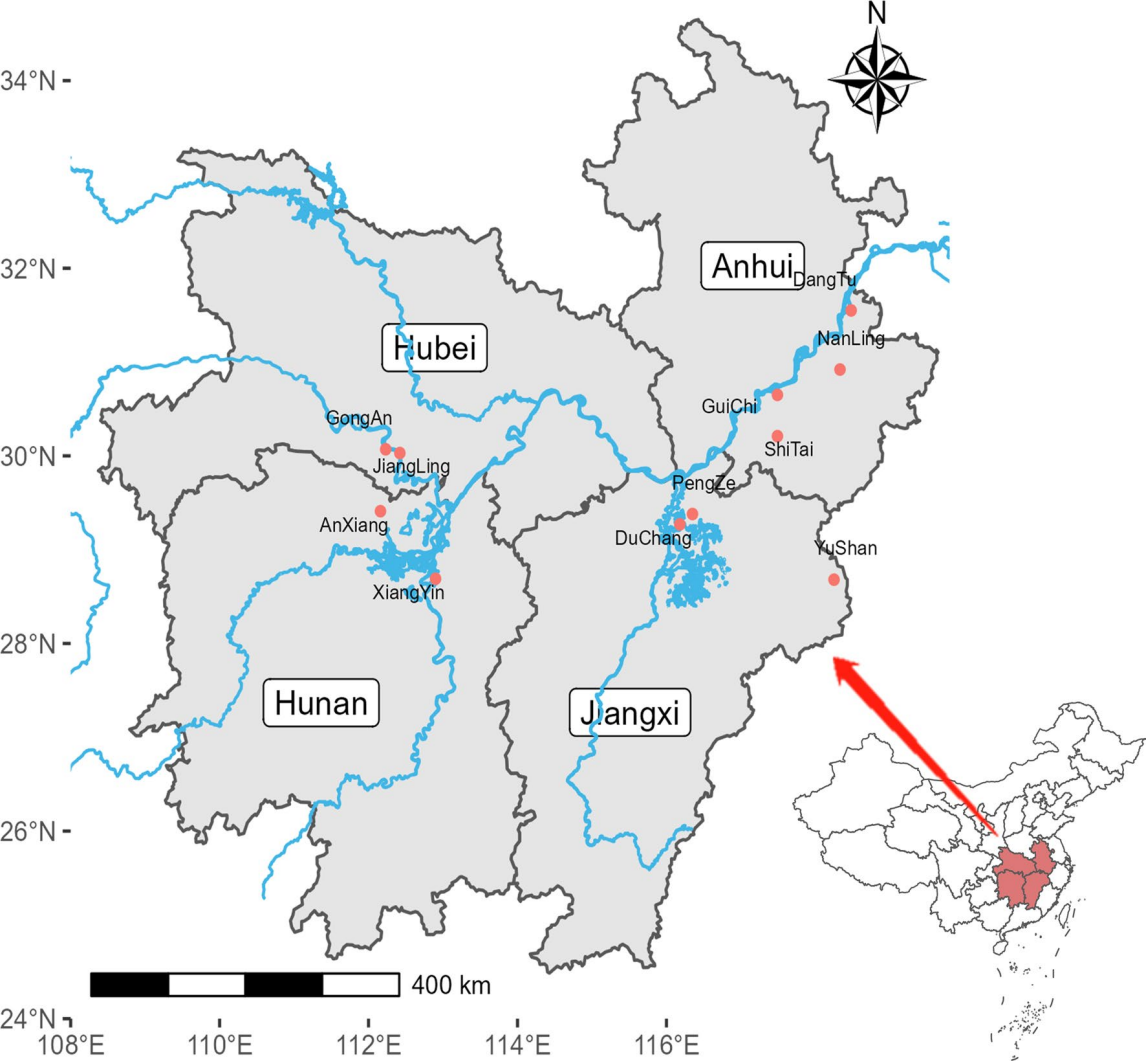
Geographical locations of the 11 sampling counties in China. All sampling counties were chosen from the four provinces in the middle and lower reaches of the Yangtze River, namely Anhui, Hunan, Hubei, and Jiangxi, specifically from those endemic counties located near water sources

Complete datasets for this study were gathered from 11 counties/cities situated at the borders of Anhui, Hunan, Hubei and Jiangxi provinces. These locations are all positioned along the middle and lower reaches of the Yangtze River (Fig. 2).

### Modeling evaluation

The fitness results of the seven machine-learning models are shown in Table 3. All of the four metrics, including R2, MSE, RMSE and MAE, suggest that the XGBoost model fitted best among seven machine-leaning models. The XGBoost model explained 73.06% of the response variables (Additional file 5).

**Table 3:** Fitness results of the seven machine-learning models with their prediction accuracy

Indicators	XGBoost	SVM	GLM	RT	RF	GBM	NNT
R^2^	0.7306	0.129	0.003	0.1811	0.5578	0.3539	0.1952
MSE	0.0041	0.0172	0.0152	0.0125	0.0068	0.0099	0.0123
RMSE	0.0641	0.1313	0.1234	0.1118	0.0822	0.0993	0.1109
MAE	0.0053	0.046	0.064	0.0408	0.028	0.0457	0.0415

*XGBoost* eXtreme gradient boosting, *SVM* support vector machine, *GLM* generalized linear model, *RT* regression tree, *RF* random forest, *GBM* gradient boosting machine, *NNT* neural network

### Marginal benefit of various interventions for overall endemic regions

After fitting the XGBoost model, the extent to which each intervention contributed to the prevalence rate was observed according to the SHAP value.

**Fig. 3:**
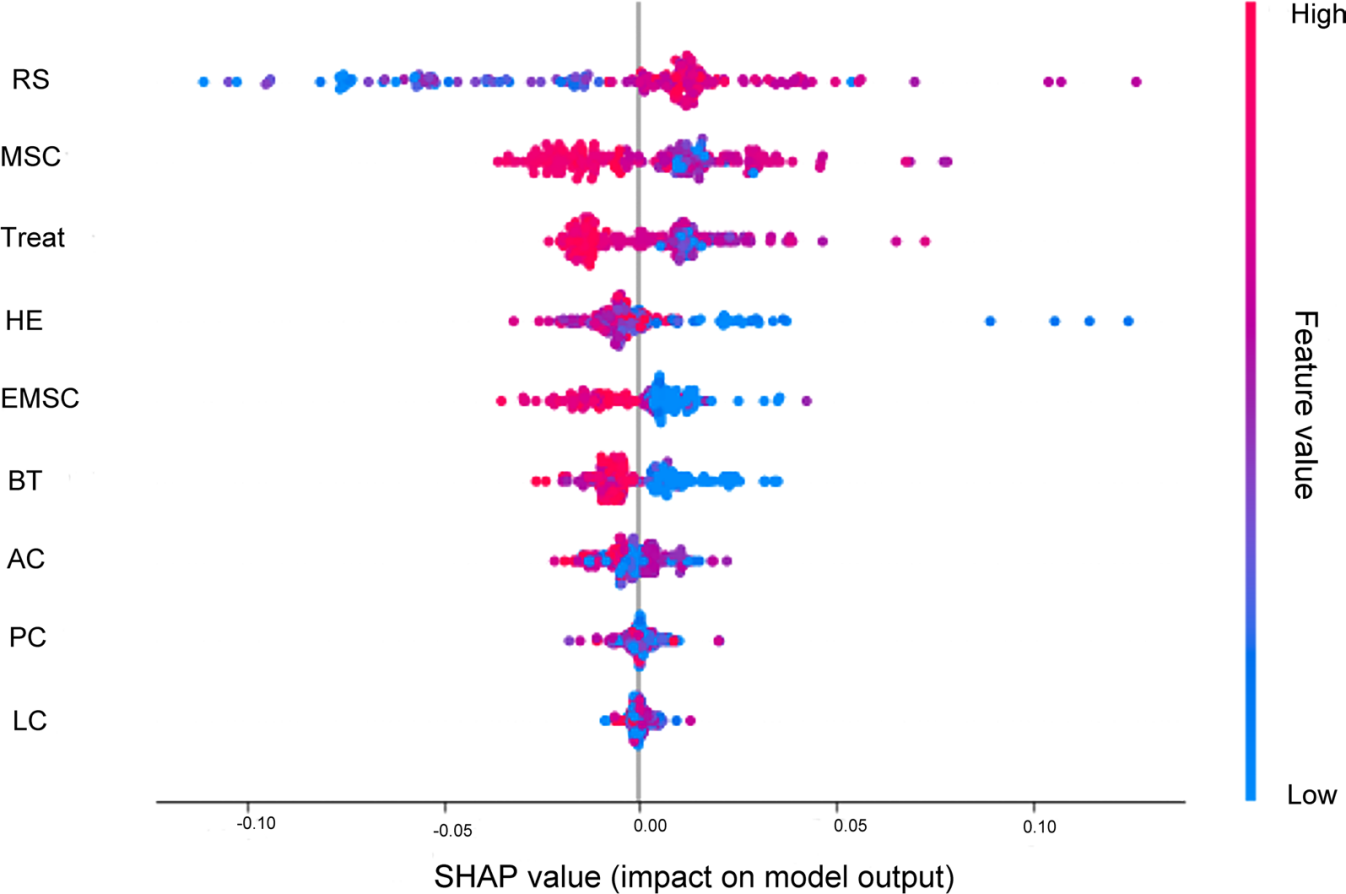
Contribution of different interventions to prevalence valued by SHAP. The vertical axis is sorted by the total sum of SHAP values for each intervention, and the horizontal axis represents the SHAP value (the distribution of the intervention's impact on the model output). Each point represents a sampling county, with overlapping points displayed vertically when their SHAP values are the same, and colors indicate the values of interventions (red corresponds to high values and blue to low values). When most of the red points are distributed on the right side, the feature is positively correlated with disease prevalence, and when they are distributed on the left side, it is negatively correlated. For example, the first row shows that higher RS costs correspond to larger SHAP values, meaning that higher RS costs lead to more cases of schistosomiasis

As shown in Fig. 3, the first row indicates that risk surveillance has the highest SHAP values, with most points clustered on the right side of the vertical axis, meaning that higher costs of risk surveillance are associated with detecting more cases of schistosomiasis. Rows two to eight show that interventions such as molluscicide for snail control, treatment, health education, environmental modification, building toilets, animal slaughter, population chemotherapy and livestock chemotherapy are effective in reducing schistosomiasis prevalence. In descending order along the vertical axis, the SHAP values of interventions gradually decrease, implying a gradual reduction in the cost-effectiveness of the interventions.

### Marginal benefit of various interventions for different regions and counties

In both types of endemic regions, namely hilly and lake-endemic areas, risk surveillance, treatment and molluscicide application for snail control emerge as the foremost interventions, occupying the top three positions in their respective hierarchies. Conversely, human and animal chemotherapy interventions are found to be the least effective across both regions.

**Fig. 4:**
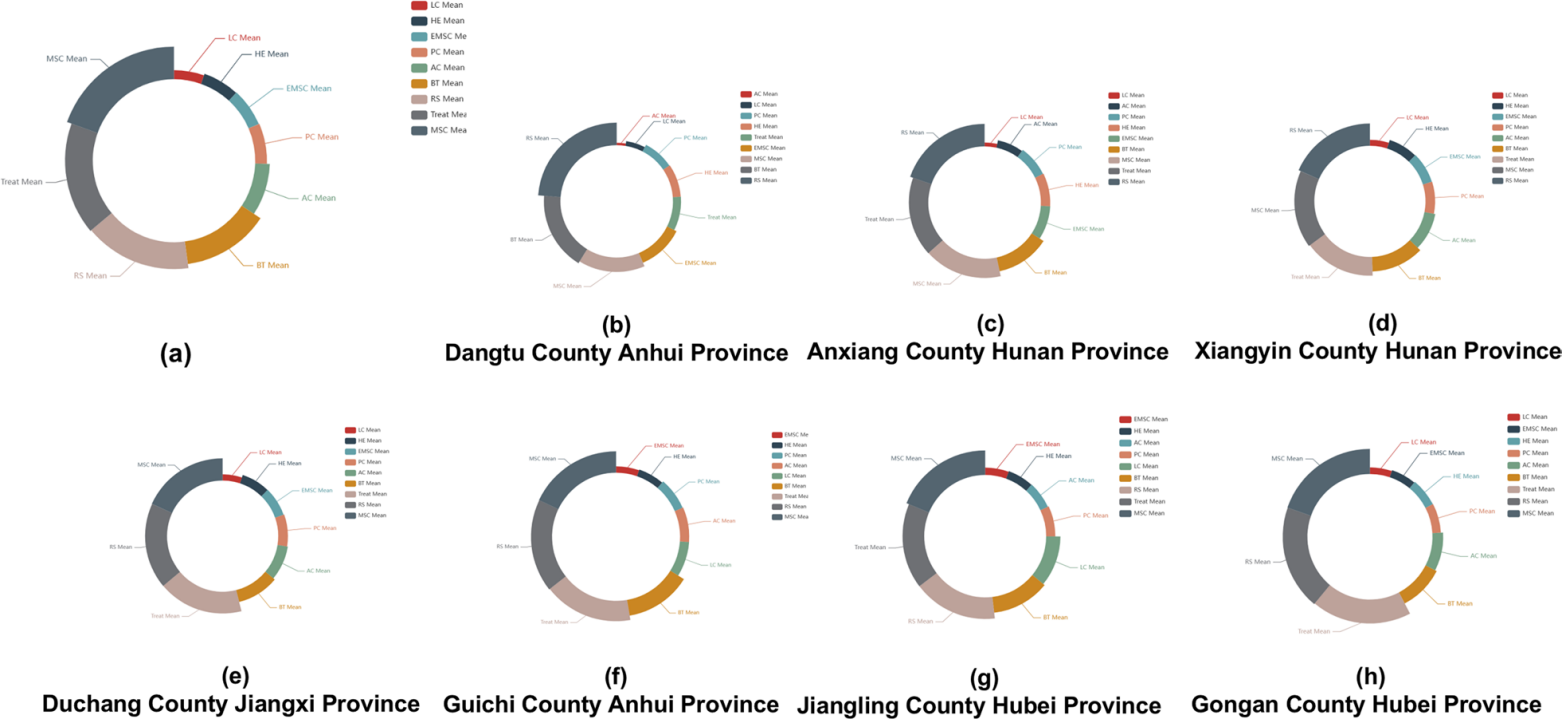
Effectiveness of different interventions to control schistosomiasis in the lake regions. Each sector arc length represents the relative effectiveness of the corresponding intervention, while the width of the sector represents the relative ranking of intervention effectiveness. The top left corner of the chart indicates the corresponding endemic area. **a** Relative importance of various measures for control of schistosomiasis in lake regions. **b**–**h** Separately illustrates the priority of different schistosomiasis interventions in Dangtu County of Anhui Province, Anxiang County of Hunan Province, Xiangyin County of Hunan Province, Duchang County of Jiangxi Province, Guichi County of Anhui Province, Jiangling County of Hubei Province and Gongan County of Hubei Province

**Fig. 5:**
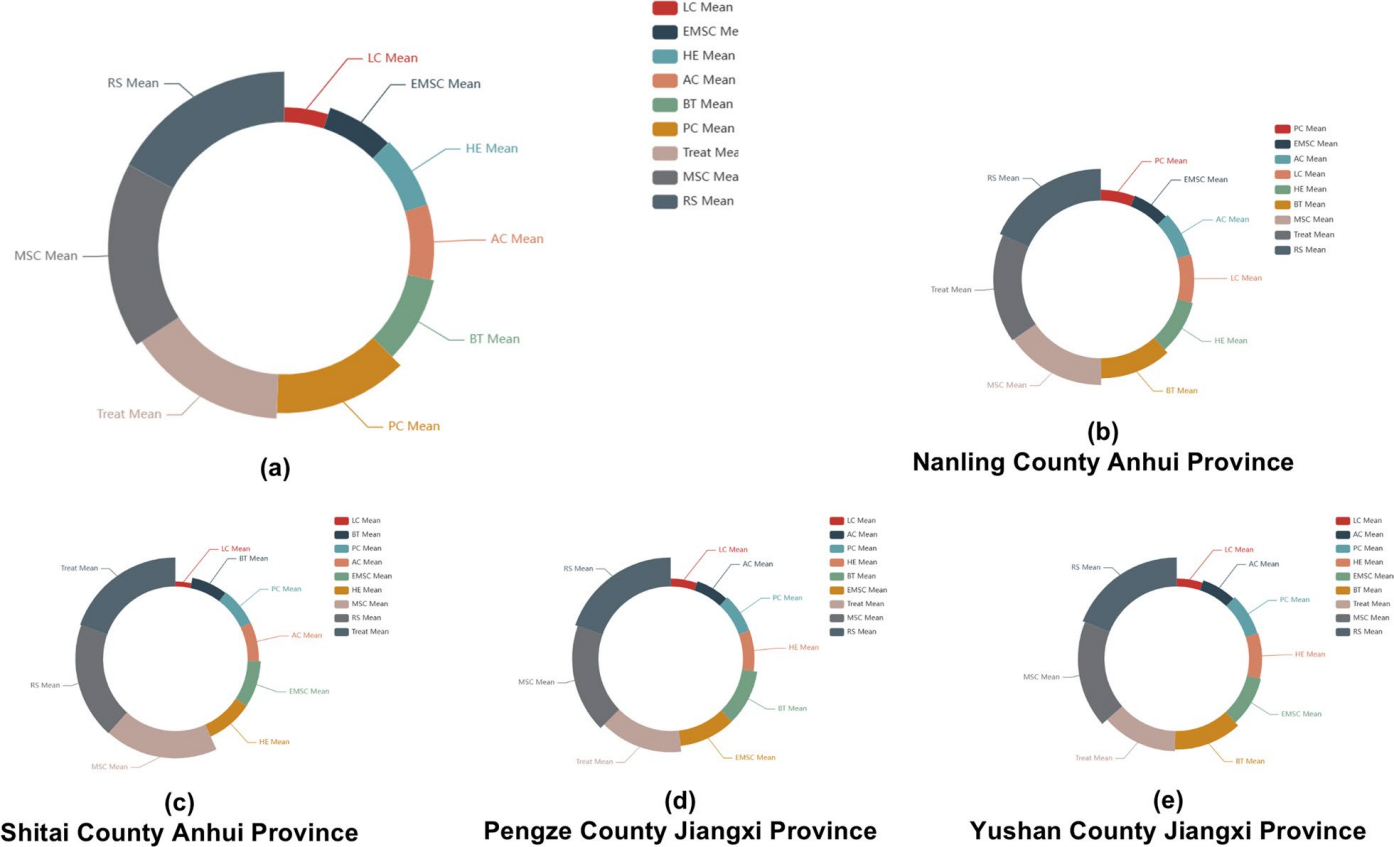
Effectiveness of different interventions to control schistosomiasis in the mountainous regions. Each sector arc length represents the relative effectiveness of the corresponding intervention, while the width of the sector represents the relative ranking of intervention effectiveness. The top left corner of the chart indicates the corresponding endemic area. **a** Priority of different measures in controlling schistosomiasis in mountainous regions where the disease is endemic. **b**–**e** Separately shows the priority of different measures in controlling schistosomiasis in Nanling County of Anhui Province, Shitai County of Anhui Province, Pengze County of Jiangxi province and Yushan County of Jiangxi province

In hilly endemic regions, risk surveillance showed the highest significance. In contrast, in lake-endemic regions, risk surveillance is relegated to the third position, implying a comparatively reduced impact when juxtaposed with molluscicide application for snail control and treatment measures. Furthermore, toilet construction secures the fourth rank in lake-endemic areas and the fifth rank in hilly endemic areas. Livestock slaughter assumes the fifth position in lake-endemic regions, while in hilly endemic areas, it occupies the sixth position within the intervention hierarchy (Figs. 4, 5).

### Optimal combinations of multi-interventions based on marginal benefits for overall endemic regions

**Fig. 6:**
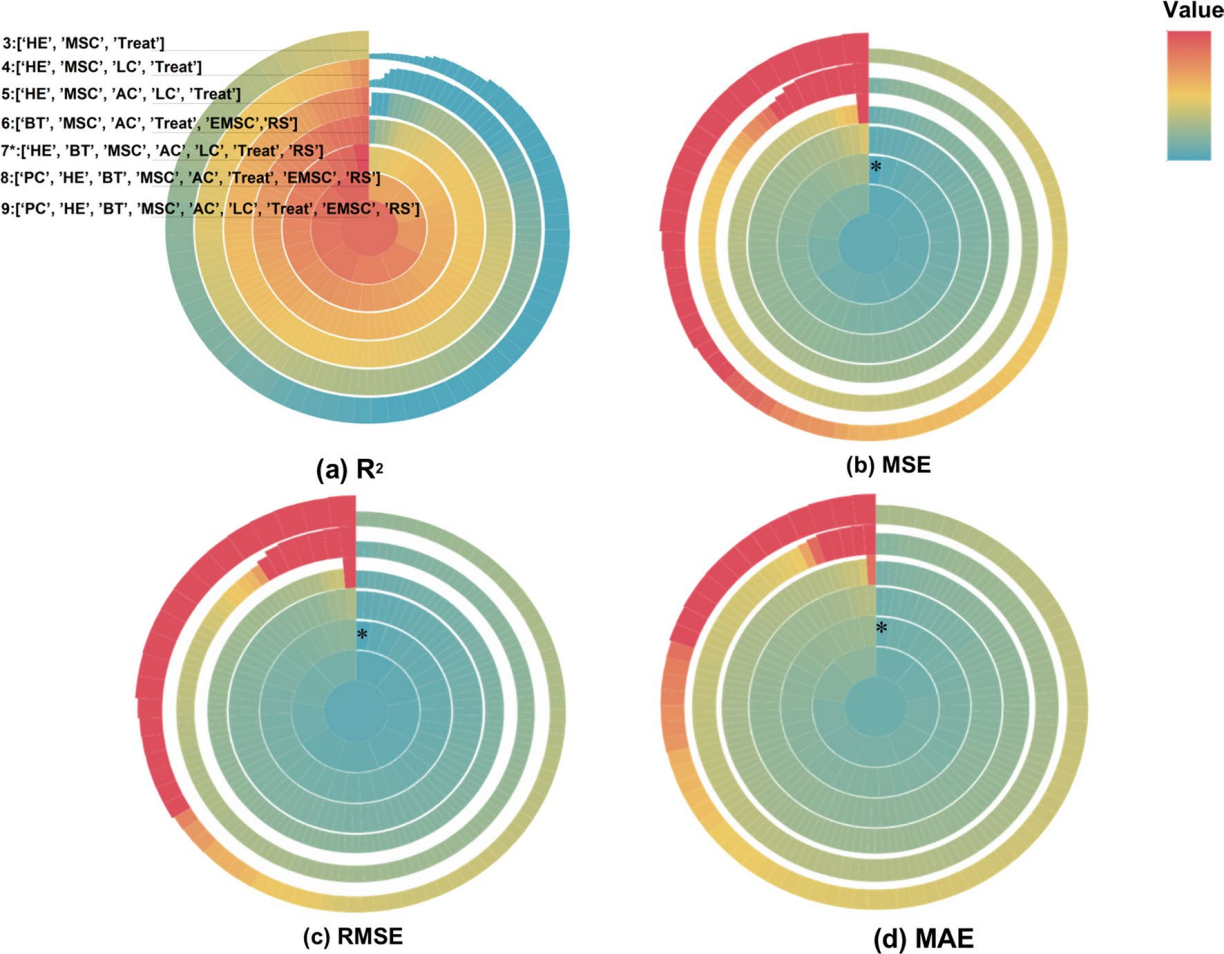
Optimal combination obtained by calculating values of R^2^, MSE, RMSE and MAE. Integrated intervention plans in this study are sorted according to the number of interventions they include. Moving from the outer to inner ring, the number of interventions included increases, ranging from three to nine. Each sector's ion rings represent a plan, and the color of the sector is represented by the values of R^2^, MSE, RMSE and MAE of the plan. **a**–**d** Separately depicts optimal combination obtained based on the values of R^2^, MSE, RMSE and MAE

Figure 6 shows that all metrics of R^2^, MSE, RMSE and MAE come to the same conclusion that the optimal combination of interventions includes health education, building toilets, controlling snails with molluscicide, animal slaughter, livestock chemotherapy, treatment and risk surveillance, resulting in the smallest residual (5.10 × 10^–7^) when fitting the prevalence. This optimal combination removed two interventions, namely mass drug administration and environmental modification to control snails. To reduce cost further, we are willing to consider suboptimal combinations that have a negligible difference in effectiveness as alternatives. When the number of interventions in the combination was reduced to six, livestock chemotherapy was removed, and when further reduced to five, toilet construction was also removed (Additional files 6, 7).

### Optimal combinations of multi-interventions based on marginal benefits for different geographical regions

**Fig. 7:**
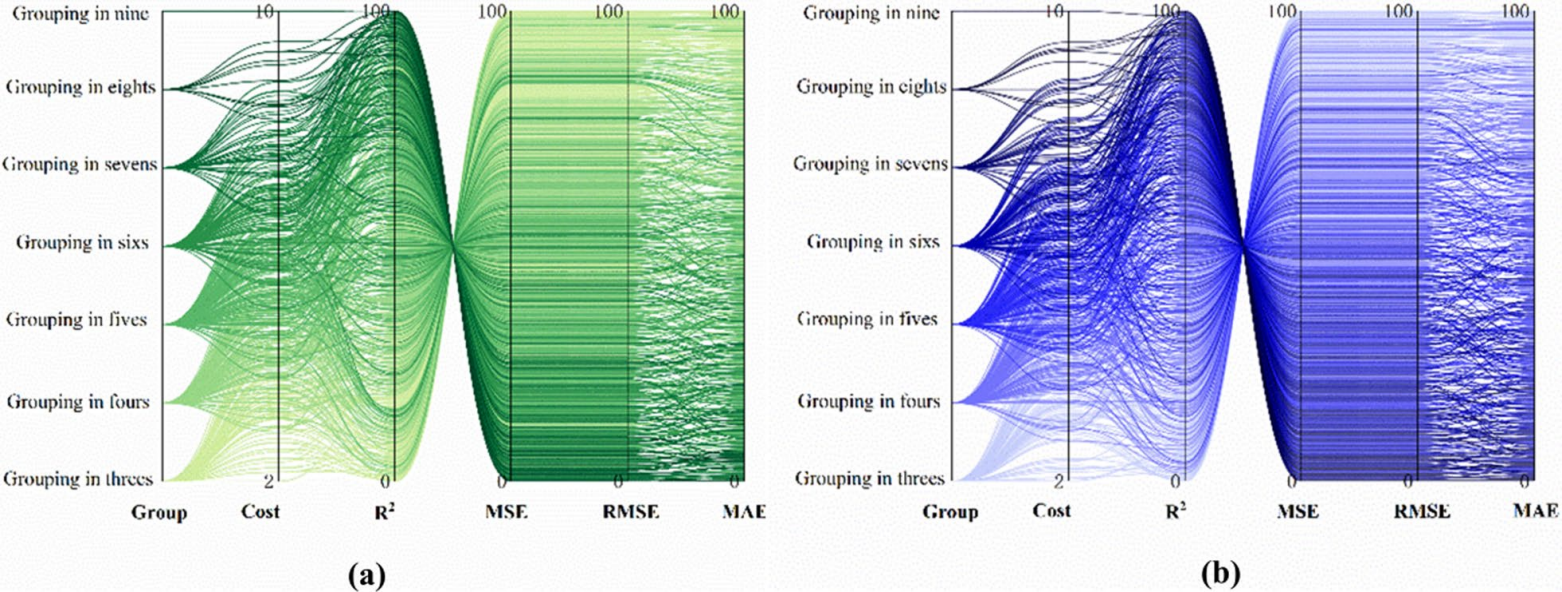
Costs of different combinations and their fit to the true values in different regions. Each line represents a different combination, with “Group” indicating the number of interventions included. The cost increases from bottom to top in the second column. The third to sixth columns show percentiles for R^2^, MSE, RMSE and MAE, with values increasing from bottom to top. Higher R^2^ indicates better effectiveness, while smaller values for MSE, RMSE and MAE also indicate better effectiveness. This is done for ease of presentation. **a**, **b** Separately depict costs of different combinations and their fit to the true values in mountainous and lake region

We applied the same approaches to identify the optimal plans for the lake and mountainous endemic regions. As depicted in Fig. 7, costs and effectiveness exhibit an inverse correlation. In the mountainous regions, the best combination involves eight interventions, including animal slaughter, health education, toilet construction, molluscicide-based snail control, population and livestock chemotherapy, treatment and risk surveillance. The two second-best combinations sequentially remove population and livestock chemotherapy and toilet construction from the optimal combination (Additional file 8).

In the lake region, the optimal combination includes seven interventions, animal slaughter, health education, toilet construction, molluscicide-based snail control, population and livestock chemotherapy, treatment and risk surveillance; the two second-best combinations further removed animal slaughter and toilet construction from the optimal combination (Additional file 8).

## Discussion

This study represents a first attempt to explore the allocation of resources for schistosomiasis control efforts during the elimination stage within counties located in the middle and lower reaches of the Yangtze River in China. We collaborated with local professional institutions to collect data from 2002 to 2021 and developed a machine learning model to analyze the priority of each intervention and the optimal combination for different regions. The results indicate that in both lake and mountainous regions, three interventions, including risk surveillance, snail control using molluscicides and treatment, play the most important role in the national schistosomiasis elimination program.

In China, the human and livestock infection rates of schistosomiasis have been extremely low in the elimination stage, so the intermediate host snail plays a pivotal role in the risk of schistosomiasis transmission [21]. Due to the frequent occurrence of flood disasters in recent years, coupled with the implementation of the Yangtze River Protection Law of the People's Republic of China [22], the total area of newly discovered and re-emergent snail habitats has increased by 110.58 hectares and 844.35 hectares from 2021 to 2022, respectively [23]. The findings of this study indicate that the effectiveness of molluscicide-based snail control, which surpasses other snail elimination methods such as environmental modification. This is evident in the SHAP-based ranking of interventions, where snail control using molluscicides consistently ranks among the top three in various endemic regions. The optimal combinations in different endemic regions all include molluscicides, while environmental modification has not been included. Similar results also were observed by other studies [24]. For example, the effectiveness of chemical snail control in controlling schistosomiasis has been confirmed, with a decreasing trend in the prevalence of schistosomiasis in the study area [25]. Moreover, counties that prioritize snail control have experienced a greater decline in infection rates compared to counties that did not adopt snail elimination interventions [26]. Many endemic areas use environmental modification for snail elimination, but the existing environmental modification technologies have high costs [27]. The primary controversies surrounding molluscicides involve concerns over environmental pollution and pollution in lake regions. Hence, it is imperative to develop new molluscicides that possess both ecological non-toxicity and exceptional snail-killing capabilities.

Despite the extremely low prevalence of schistosomiasis in China at present, early-stage surveillance remains insufficient, leading to challenges in achieving timely disease diagnosis and intervention. As a result, China continues to identify about 1000 new cases of advanced schistosomiasis each year due to previous exposure [23]. Additionally, based on the experiences of eliminating malaria (by 2021) and lymphatic filariasis (by 2007) in China, sustained risk surveillance plays an important role in the elimination of the disease as it can help to identify, isolate and control infected individuals, reduce the risk of transmission and improve the efficiency of elimination [28].

To advance the process of the national eliminating schistosomiasis program in China, it is essential to strengthen surveillance strategies. This includes intensifying the surveillance of changes in snail-inhabited areas and the infection rates of humans, cattle and snails and simultaneously conducting explorations of potential high-risk regions. The results of this study demonstrate that risk surveillance is a pivotal intervention for the elimination of schistosomiasis [29]. This conclusion is supported by two outcomes from the study. First, among all intervention measures, risk surveillance exhibits the highest SHAP value. Second, optimal combinations from the study in both lake and mountainous regions have included the intervention of risk surveillance. This study also provides a reference for establishing surveillance priorities. The determination of surveillance focus is guided by assessing the marginal benefits derived from interventions targeting humans, cattle and snails in different endemic regions.

Once high-risk areas have been identified through surveillance, integrated strategies should be carried out based on the reference of the optimal intervention combinations. The results of this research reveal significant variations in the optimal integrated strategies between lake- and mountain-endemic regions. These discrepancies are likely attributed to the geographical morphology, socio-cultural factors and transmission characteristics of schistosomiasis within these areas [30–33]. For example, in regions with dense vegetation, molluscicides can easily adhere to plant leaves, resulting in limited efficacy in snail elimination. In such scenarios, environmental modification yields better outcomes for snail elimination [30]. By analyzing data from different endemic regions between 2002 and 2020, this study presents optimized integrated strategies for areas with varying geographical conditions, socio-cultural aspects and transmission characteristics. These findings contribute to the advancement of schistosomiasis elimination efforts in China.

This study is subject to certain limitations. First, our reliance on prevalence as the indication for estimating marginal benefit restricts our capacity to precisely quantify the potential impact of interventions in interrupting schistosomiasis transmission. Second, it is essential to recognize that the extended data collection period in this study created complications due to administrative changes like mergers and divisions of institutions. These changes led to missing data, which could affect the study's completeness. Third, our research is geographically delimited to four distinct provinces within China, specifically Hubei, Hunan, Anhui and Jiangxi. Consequently, there is a pertinent imperative for subsequent investigations encompassing a broader array of endemic regions, diverse transmission dynamics and a spectrum of disease types to foster a more comprehensive understanding of the subject matter.

## Conclusions

This study has made significant contributions to understanding resource allocation and surveillance strategies for schistosomiasis elimination in China. The research emphasizes the importance of risk surveillance, adaptable to diverse geographical and socio-cultural contexts. Additionally, the study shows that chemical molluscicides can be more effective than environmental modifications, especially in densely vegetated areas. This study offers insights for global schistosomiasis control and informs China’s public health policies and interventions.

### Supplementary Information

**Additional file 1: **Brief introduction of machine learning model

**Additional file 2: **Parameters of XGBoost. **Table S1.** XGBoost model parameters obtained through grid search.

**Additional file 3: **Brief introduction of marginal benefit combined with schistosomiasis elimination. **Fig. S1.** Marginal cost and marginal benefit.

**Additional file 4**: Lag parameters of interventions. **Fig. S2.** Visualizing the correlation between costs of interventions and prevalence. **Table S2.** Correlation between the costs of interventions and prevalence. **Fig. S3.** Visualizing the correlation between health education with a five-year lag and prevalence. **Table S3. **Correlation between health education with a 5-year lag and disease prevalence. **Fig. S4.** Visualizing the correlation between environmental modification for snail control with a 5-year lag and prevalence. **Table S4.** Correlation between environmental modification for snail control with a 5-year lag and the prevalence.

**Additional file 5**: Comparing the prediction accuracy of seven machine learning models. **Fig. S5.** Visualizing the predictive accuracy of different models.

**Additional file 6**: Optimal combinations of different size combinations. **Fig. S6.** Visualization of optimal combinations for different sizes of groups.

**Additional file 7**: Priority of the optimal combination. **Fig. S7.** Contribution ratio of different interventions to prevalence valued by SHAP value in optimal group.

**Additional file 8**: Optimal combinations of different size combinations in lake and mountainous region. **Table S5. **Optimal combinations for different sizes of groups in lake and hill type endemic area.

## Data Availability

The following supporting information can be downloaded at: https://github.com/liqin0724/optimal_schistosomiasis_model.git.

## Abbreviations

ML: Machine learning
RS: Risk surveillance
MSC: Molluscicide for snail control
Treat: Treatment
PC: Population chemotherapy
BT: Building toilets
EMSC: Environmental modification for snail control
AR: Animal slaughter
HE: Health education
LC: Livestock chemotherapy
XGBoost: EXtreme gradient boosting
SVM: Support vector machine
GLM: Generalized linear model
RT: Regression tree
RF: Random forest
GBM: Gradient boosting machine
NNT: Neural network
