# Supplementary material for: Optimized strategy for schistosomiasis elimination: results from marginal benefit modeling
Source: Parasit Vectors. 2023 Nov 15;16:419. doi: 10.1186/s13071-023-06001-x (PMC10652544; doi:10.1186/s13071-023-06001-x)
Supplement: Supplementary file 1 — Additional file 1: Brief introduction of machine learning model [file 13071_2023_6001_MOESM1_ESM.docx]

**Neural networks**

A neural network model is a mathematical model that simulates the function of biological neurons to receive, converge, analyze, transmit and process information^[1]^. The external information is fed into the model and then weighted and aggregated, and this weight varies depending on the connection weights of the different neurons. Next, the external information is mapped onto the activation states through the neuron activation function, which ultimately gives the final activity value of the neuron^[2]^. This is an artificial neuron model. Different types of neurons have different activation functions. The activation functions are continuously derivable non-linear functions, and the derivable activation functions can be used directly to learn the network parameters using numerical optimization methods; secondly, the activation functions and their derivatives should be as simple as possible to help improve the efficiency of network computation. the range of values of the derivative function of the activation function should be in a suitable range, otherwise it will affect the efficiency and stability of the model training^[3]^. The activation function is generally a monotonically increasing function, and in recent years some studies have also allowed the activation function to fluctuate over a small number of intervals. Commonly used activation functions are: S-type functions, ramp functions, and composite functions^[4]^. Composite functions as activation functions are generally used in large neural network models. The basic unit that makes up an artificial neural network is the neuron. Neural networks are formed by the directed connections between different neurons. When constructing a neural network the focus is on: the activation function, the topology of the network, and the learning algorithm. Artificial neural networks are information processing networks consisting of many neurons with a parallel distribution structure. Gradient descent in parameter space is achieved using gradient information. By training a neural network, we are minimising a loss function^[5]^. The value of this loss function measures how close the performance of our network is to perfection on a given data set. When training a neural network model, the current loss is calculated in each iteration with respect to the parameter gradient and the parameters are updated.

**Gradient boosting machine**

The GBM (Gradient Boosting Machine) algorithm is a type of Boosting (boosting) algorithm. The main idea is that the GBM is a modified version of a decision tree that uses structural scoring, gain calculation and progressive refinement approximation to build a set of optimal and satisfactory tree structures by comparative analysis of each variable brought into the model^[6]^. GBM can handle missing data, is not disturbed by noisy data, has high accuracy when fitting complex non-linear models, and is relatively fast to computer^[7]^. The disadvantage is that GBM is serially generated for multiple weak learners and has more parameters to set.

When training the gradient boosting machine, unlike a neural network, the gradient boosting machine generates multiple weak learners serially, each with the objective of fitting the negative gradient of the loss function of the previously accumulated model, so that the cumulative model loss after the addition of the weak learner is reduced in the direction of the negative gradient^[8]^. and it linearly combines the base learners with different weights so that the top performing learners are reused. In this way a weak learner is generated in each iteration and this new learner fits the loss function with respect to the gradient of the previous cumulative model, and this weak learner is then added to the cumulative model to gradually reduce the loss of the cumulative model, i.e. to reduce the loss by fitting a new function using the gradient.

**XGBoost model**

The basic principle of XGBoost is similar to that of the gradient boosting machine, but there are many modifications to XGBoost^[9]^. The weak learner used by XGBoost is the decision tree. When the decision tree is used as a weak learner, features with large variance between single decision trees enhance the generalisation performance of the model. (a) Compared to the first-order derivatives of the gradient boosting model, XGBoost does a second-order Taylor expansion of the loss function, applying both first- and second-order derivatives to make the loss function more accurate. (b) XGBoost adds a regular term to the loss function for controlling the complexity of the model. In terms of Bias-variance tradeoff, the canonical term reduces the variance of the model, making the learned model simpler and avoiding overfitting. XGBoost borrows from random forests and supports column sampling, which not only reduces overfitting, but also reduces computation. Before training the XGBoost model, the data is pre-sorted and saved as a block structure, which is used repeatedly in later iterations to greatly reduce the computational effort, and the block structure also makes the parallel structure possible. That is, when splitting the nodes, the gain of each feature needs to be calculated and the feature with the greatest gain is finally selected for splitting, then the gain calculation of each feature can be done in multiple threads.

**The regression tree**

The advantages of regression tree models are that they are easy to understand and interpret, easy to visualise and analyse, and extract the decision rules of the model^[10]^. Can produce feasible and effective results for large data sources in a relatively short time; can handle interactions between parametric or non-parametric features^[11]^; disadvantages are difficulty in handling missing data; prone to overfitting. Tends to learn in the direction of deeper trees; ignores interrelationships in the dataset; does not support online learning and decision trees need to be rebuilt when new samples arrive.

**The support vector machine**

The vector machine model is a powerful regression model that uses kernel functions to convert non-separable problems into more easily solvable separable problems^[12]^. Vector machine models can easily handle non-linear relationships between features. A good way to develop models is to train SVMs with radial basis function kernels to quantify the gains that can be made from non-linear models^[13]^. The non-linear approach can provide a more robust model at the cost of ease of interpretation of which features are influencing the model. The disadvantages are reduced efficiency when there are many observed samples; no general solution to the non-linear problem; and sensitivity to missing data.

The relationship between features in the data is often non-linear, so in these cases using a model such as an SVM is often a more appropriate choice. SVM is a powerful regression and classification model that uses kernel functions to convert non-separable problems into more easily solvable separable problems^[14]^. SVM can be used to perform both linear and non-linear regression, depending on the kernel function used. A good way to develop models is to train linear SVMs and SVMs with a radial basis function kernel (a generic non-linear type of SVM) to quantify the gains, if any, that can be obtained from non-linear models. The non-linear approach can provide a more robust model, but at the cost of ease of interpretation of which features are influencing the model, a trade-off mentioned in the introduction.

**Random forest**

A random forest is a type of cluster classification model. A random forest is a forest built in a random way, consisting of many decision trees, and each decision tree is unrelated to each other. After the random forest model is obtained, each decision tree in the random forest is judged separately when a new sample enters. The bagging aggregation strategy is relatively simple, and for classification problems the voting method is usually used, with the most votes category or one of the categories being the final model output. For regression, simple averaging is usually used, where the regression results from T weak learners are arithmetically averaged to give the final model output Random Forest uses CART decision trees as the weak learners. The advantages of the model are Advantages: uses an integrated algorithm with better accuracy than a single decision tree algorithm; performs well on the test set and is able to reduce the risk of overfitting and improve noise immunity due to two randomities: random samples and random features^[15]^; due to the combination of trees, makes the random forest able to handle non-linear data, which itself is a non-linear classification and fitting model; it is able to handle very high dimensional data and does not have to do feature selection and is adaptable to the data^[16]^; it can handle both discrete and continuous data, and the data does not need to be normalized; it is fast to train and can be applied to large-scale datasets; can handle default values (by grouping them into one category); for out-of-set data, unbiased estimates of the true error can be obtained during model generation without loss of training data^[17]^; in the training data, it is possible to detect inter-feature It is possible to detect interactions between features in the training data and to obtain the importance of the features; each tree can be generated independently and simultaneously, making it easy to parallelise^[18]^; simple to implement, high accuracy and strong resistance to overfitting; disadvantage: easy to overfit in noisy classification and regression problems

**Generalised linear model**

The generalised linear model (GLM) is an extension of the linear model, which establishes the relationship between the mathematical expectation of the response variable and a linear combination of predictor variables through a link function^[19]^. It is characterised by the fact that the natural measure of the data is not forced to change and the data can have a non-linear and non-constant variance structure^[20]^. It is a development of linear models in the study of non-normal distributions of response values and the concise and direct linear transformation of non-linear models.

**Reference**

[1] MÜLLER M G, PAPADIMITRIOU C H, MAASS W, et al. A Model for Structured Information Representation in Neural Networks of the Brain. eNeuro. 2020; 7(3).

[2] KRIEGESKORTE N, GOLAN T. Neural network models and deep learning. Curr Biol. 2019; 29(7): R231-r6.

[3] STANEV V, OSES C, KUSNE A G, et al. Machine learning modeling of superconducting critical temperature. npj Computational Materials. 2018; 4(1): 29.

[4] HENNEQUIN G. Neural networks subtract and conquer. Elife. 2017; 6.

[5] YANG Y, WANG T, WOOLARD J P, et al. Guaranteed approximation error estimation of neural networks and model modification. Neural Netw. 2022; 151: 61-9.

[6] NATEKIN A, KNOLL A. Gradient boosting machines, a tutorial. Frontiers in Neurorobotics. 2013; 7.

[7] YANG S, WU J, DU Y, et al. Ensemble Learning for Short-Term Traffic Prediction Based on Gradient Boosting Machine. Journal of Sensors. 2017; 2017: 7074143.

[8] GU T, DOLAN-GAVITT B, GARG S. BadNets: Identifying Vulnerabilities in the Machine Learning Model Supply Chain. ArXiv. 2017; abs/1708.06733.

[9] CHEN T, GUESTRIN C. XGBoost: A Scalable Tree Boosting System. Proceedings of the 22nd ACM SIGKDD International Conference on Knowledge Discovery and Data Mining. 2016.

[10] KRZYWINSKI M, ALTMAN N. Classification and regression trees. Nature Methods. 2017; 14(8): 757-8.

[11] MARSHALL R J. The use of classification and regression trees in clinical epidemiology. Journal of Clinical Epidemiology. 2001; 54(6): 603-9.

[12] NOBLE W S. What is a support vector machine? Nat Biotechnol. 2006; 24(12): 1565-7.

[13] WILLIAMS P, LI S, FENG J, et al. A geometrical method to improve performance of the support vector machine. IEEE Trans Neural Netw. 2007; 18(3): 942-7.

[14] CHAPELLE O. Training a support vector machine in the primal. Neural Comput. 2007; 19(5): 1155-78.

[15] RIGATTI S J. Random Forest. J Insur Med. 2017; 47(1): 31-9.

[16] YI Y, SUN D, LI P, et al. Unsupervised random forest for affinity estimation. Comput Vis Media (Beijing). 2022; 8(2): 257-72.

[17] SVETNIK V, LIAW A, TONG C, et al. Random forest: a classification and regression tool for compound classification and QSAR modeling. J Chem Inf Comput Sci. 2003; 43(6): 1947-58.

[18] SAVARGIV M, MASOUMI B, KEYVANPOUR M R. A New Random Forest Algorithm Based on Learning Automata. Comput Intell Neurosci. 2021; 2021: 5572781.

[19] CHEN L W, FINE J P, BAIR E, et al. Semiparametric analysis of a generalized linear model with multiple covariates subject to detection limits. Stat Med. 2022; 41(24): 4791-808.

[20] PEUGH J L, BEAL S J, MCGRADY M E, et al. Analyzing discontinuities in longitudinal count data: A multilevel generalized linear mixed model. Psychol Methods. 2021; 26(4): 375-97.
