# Supplementary material for: Optimized strategy for schistosomiasis elimination: results from marginal benefit modeling
Source: Parasit Vectors. 2023 Nov 15;16:419. doi: 10.1186/s13071-023-06001-x (PMC10652544; doi:10.1186/s13071-023-06001-x)
Supplement: Supplementary file 2 — Additional file 2: Parameters of XGBoost. Table S1. XGBoost model parameters obtained through grid search. [file 13071_2023_6001_MOESM2_ESM.docx]

After performing grid search, the optimal parameters for the model were determined to be: subsample of 0.7, 'reg_lambda' of 0.0001, 'reg_alpha' of 1, 100 'n_estimators', a 'min_child_weight' of 5, 'max_depth' of 4, 'learning_rate' of 0.2, a 'gamma' of 0.4, and a 'colsample_bytree' of 0.6. The roles of these parameters are summarized in the table below.

Table S1 XGBoost model parameters obtained through grid search

| Parameter name | Meaning | Value |
| --- | --- | --- |
| max_depth | This is the number of weak learners to be fitted, the larger the value, the more complex the model and the more likely it is to be over-fitted | 4 |
| n_estimators | the number of weak learners to be fitted, the higher the value, the more complex the model and the more likely it is to be over-fitted | 100 |
| learning_rate | The step size used in the update shrinks to prevent over-fitting. | 0.2 |
| gamma | The minimum loss required to divide further on the leaf nodes of the tree is reduced. the larger the gamma, the more conservative the algorithm. | 0.4 |
| reg_alpha | L1 regularisation is used to penalize the number of leaves and to prevent over-fitting | 1 |
| reg_lambda | L2 regularisation is used to penalize the scores of the leaf nodes | 0.0001 |
| min_child_weight | The sum of the weights of the minimum samples, which will no longer split if the sum of the weights of the samples is less than this value during the growth of the tree; the larger the min_child_weight, the more conservative the algorithm will be. | 5 |
| colsample_bytree | A sampling rate of features per tree used to prevent over-fitting | 0.6 |
| subsample | A sampling rate of the training set to sample instances, to prevent overfitting | 0.7 |
