# Supplementary material for: Optimized strategy for schistosomiasis elimination: results from marginal benefit modeling
Source: Parasit Vectors. 2023 Nov 15;16:419. doi: 10.1186/s13071-023-06001-x (PMC10652544; doi:10.1186/s13071-023-06001-x)
Supplement: Supplementary file 3 — Additional file 3: Brief introduction of marginal benefit combined with schistosomiasis elimination. Fig. S1. Marginal cost and marginal benefit. [file 13071_2023_6001_MOESM3_ESM.docx]

The marginal benefit method is an economic evaluation method that can transfer resources between different plans to maximize the benefits of available resources, that is, allocate more resources to high-risk groups or more beneficial prevention and control measures to increase total benefits without changing the total resources. For example, a small-scale disease screening for high-risk groups that discovers all positive cases has lower costs and higher benefits, while discovering all positive cases in large-scale screening activities is more expensive and less beneficial. Therefore, on the basis of large-scale screening, when using more targeted and high-tech screening methods to detect new cases in high-risk groups, there will be higher marginal costs and benefits. Compared with the overall demand for screening evaluation, the new technology screening is more effective and helps to improve decision-making. For example, in response to a new influenza pandemic, the conventional measure is to vaccinate a large population, and the additional screening and control of super-spreaders are marginal inputs, which produce marginal benefits. There is a trade-off between disease loss and prevention and control costs. As the intensity of prevention and control increases, the total cost of prevention and control increases, and the total disease loss decreases. After the intensity of prevention and control increases, the infection rate decreases, and it becomes more difficult to detect cases. The benefit of prevention and control obtained by the same prevention and control cost decreases. Therefore, the required prevention and control cost gradually increases, that is, the marginal prevention and control cost increases, and the marginal disease loss decreases. At this time, the decision goal of prevention and control should be to maximize the benefit of prevention and control, that is, to minimize the sum of disease loss and prevention and control cost, and the reduced disease loss is the benefit of prevention and control. That is Max prevention and control benefit = Min (disease loss + prevention and control cost). To solve this maximization problem, the first-order condition of the optimal policy is that the marginal benefit equals its marginal cost, that is, marginal disease loss = marginal prevention and control cost. The net benefit is defined as the difference between the area under the marginal disease loss curve and the marginal prevention and control cost curve. When the marginal disease loss is greater than the marginal prevention and control cost, the net benefit of intervention increases; when the marginal disease loss is less than the marginal prevention and control cost, the net benefit decreases; when the marginal prevention and control cost is equal to the marginal disease loss, the net benefit is maximized. When the research object is expanded to a large-scale project consisting of many decisions, using marginal analysis as the basis for each decision to obtain the total benefit is the maximum benefit of the large-scale project.


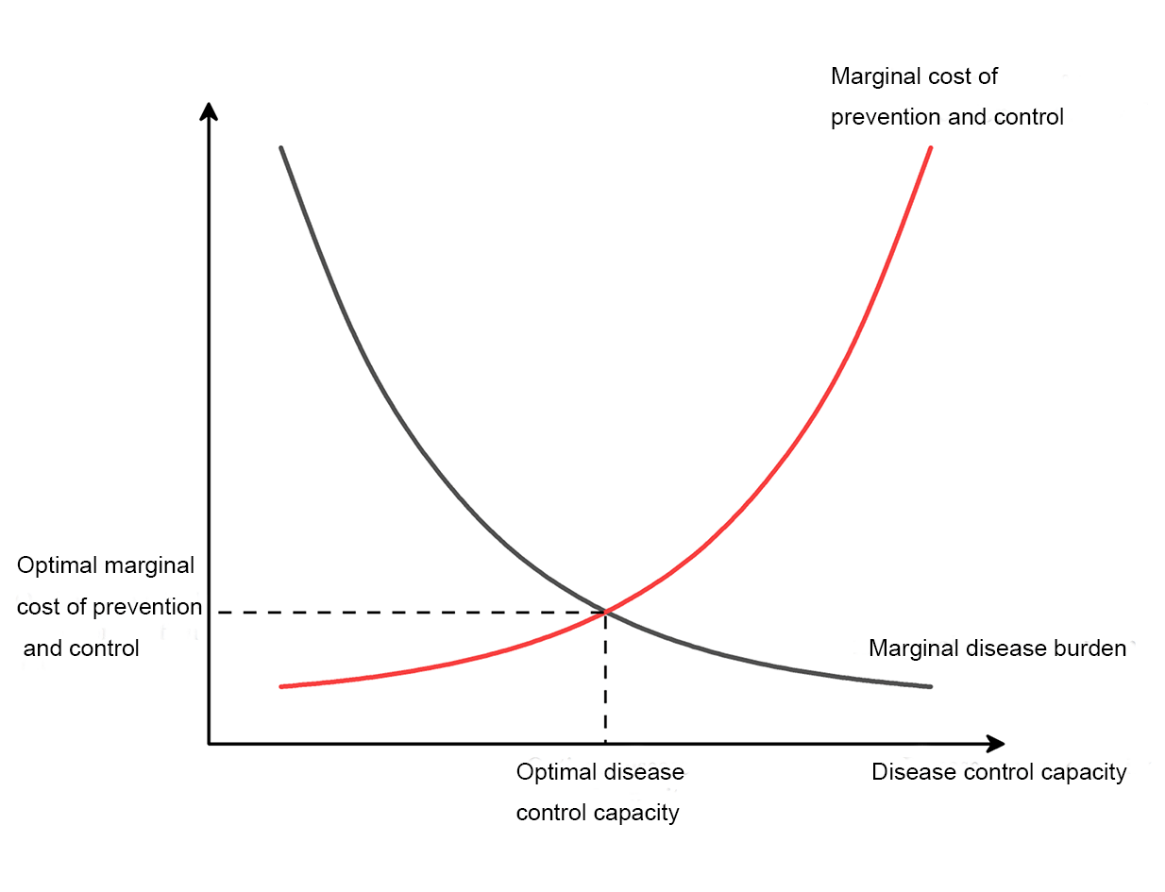


**Figure S1** Marginal cost and marginal benefit
