# Supplementary material for: Optimized strategy for schistosomiasis elimination: results from marginal benefit modeling
Source: Parasit Vectors. 2023 Nov 15;16:419. doi: 10.1186/s13071-023-06001-x (PMC10652544; doi:10.1186/s13071-023-06001-x)
Supplement: Supplementary file 4 — Additional file 4: Lag parameters of interventions. Fig. S2. Visualizing the correlation between costs of interventions and prevalence. Table S2. Correlation between the costs of interventions and prevalence. Fig. S3. Visualizing the correlation between health education with a five-year lag and prevalence. Table S3. Correlation between health education with a 5-year lag and disease prevalence. Fig. S4. Visualizing the correlation between environmental modification for snail control with a 5-year lag and prevalence. Table S4. Correlation between environmental modification for snail control with a 5-year lag and the prevalence. [file 13071_2023_6001_MOESM4_ESM.docx]

The standard for determining the lag parameter in this study is: the correlation coefficient conforms to epidemiological common sense in terms of positive or negative signs, and the absolute value is greater than 0.05.


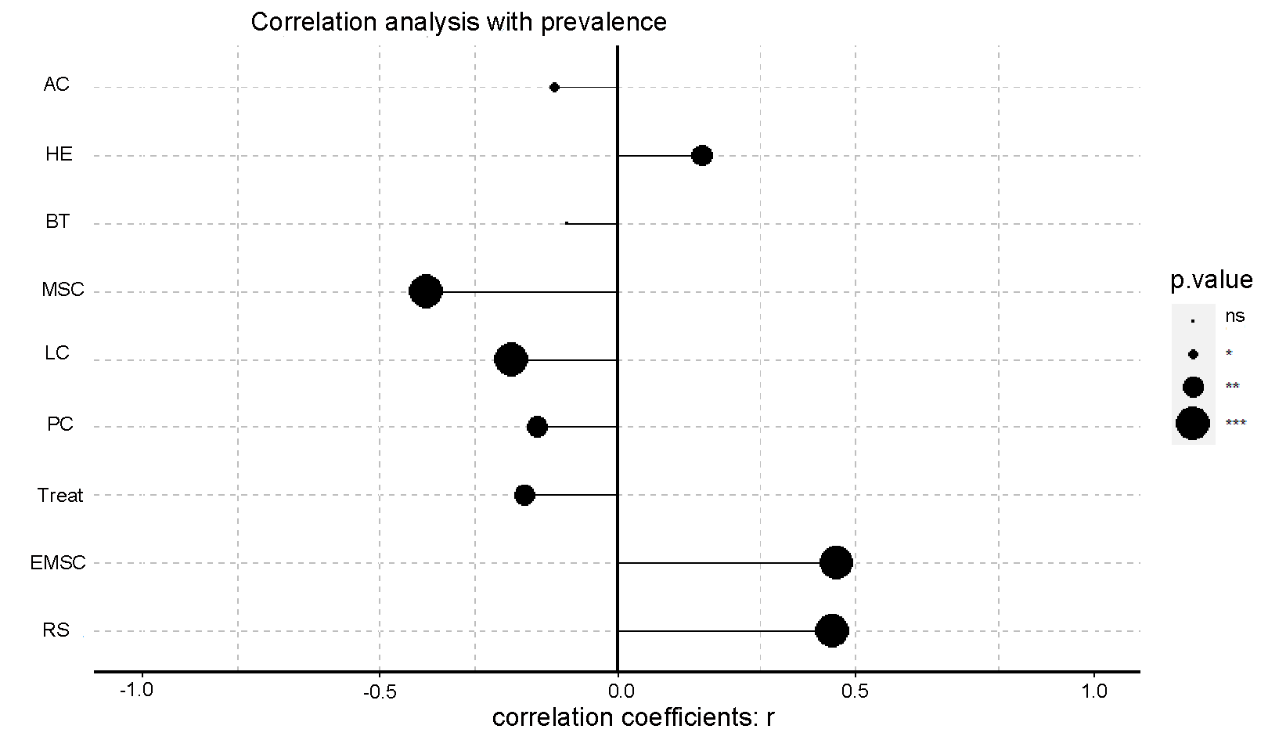


**Figure S2** Visualizing the correlation between costs of interventions and prevalence (Risk surveillance (RS), Molluscicide for snail control (MSC), Treatment (Treat), Population chemotherapy (PC), Building toilets (BT), Environmental modification for snail control (EMSC), Animal culling (AC), Health education (HE), and Livestock chemotherapy (LC). The figure displays the correlation coefficient on the horizontal axis, indicating both its magnitude and direction. The vertical axis arranges the variables in the order they were inputted, while P-values are denoted by asterisks and dots, <0.01:***, <0.05:**, <0.1:*, ≥0.1:ns.)

**Table S2** The correlation between the costs of interventions and prevalence

| Variable 1 | Variable 2 | Correlation coefficient | P value |
| --- | --- | --- | --- |
| Prevalence | AC | -0.13 | 0.09 |
| Prevalence | HE | 0.18 | 0.03 |
| Prevalence | BT | -0.11 | 0.18 |
| Prevalence | MSC | -0.4 | 0.00 |
| Prevalence | LC | -0.22 | 0.00 |
| Prevalence | PC | -0.17 | 0.03 |
| Prevalence | Treat | -0.2 | 0.01 |
| Prevalence | EMSC | 0.46 | 0.00 |
| Prevalence | RS | 0.45 | 0.00 |


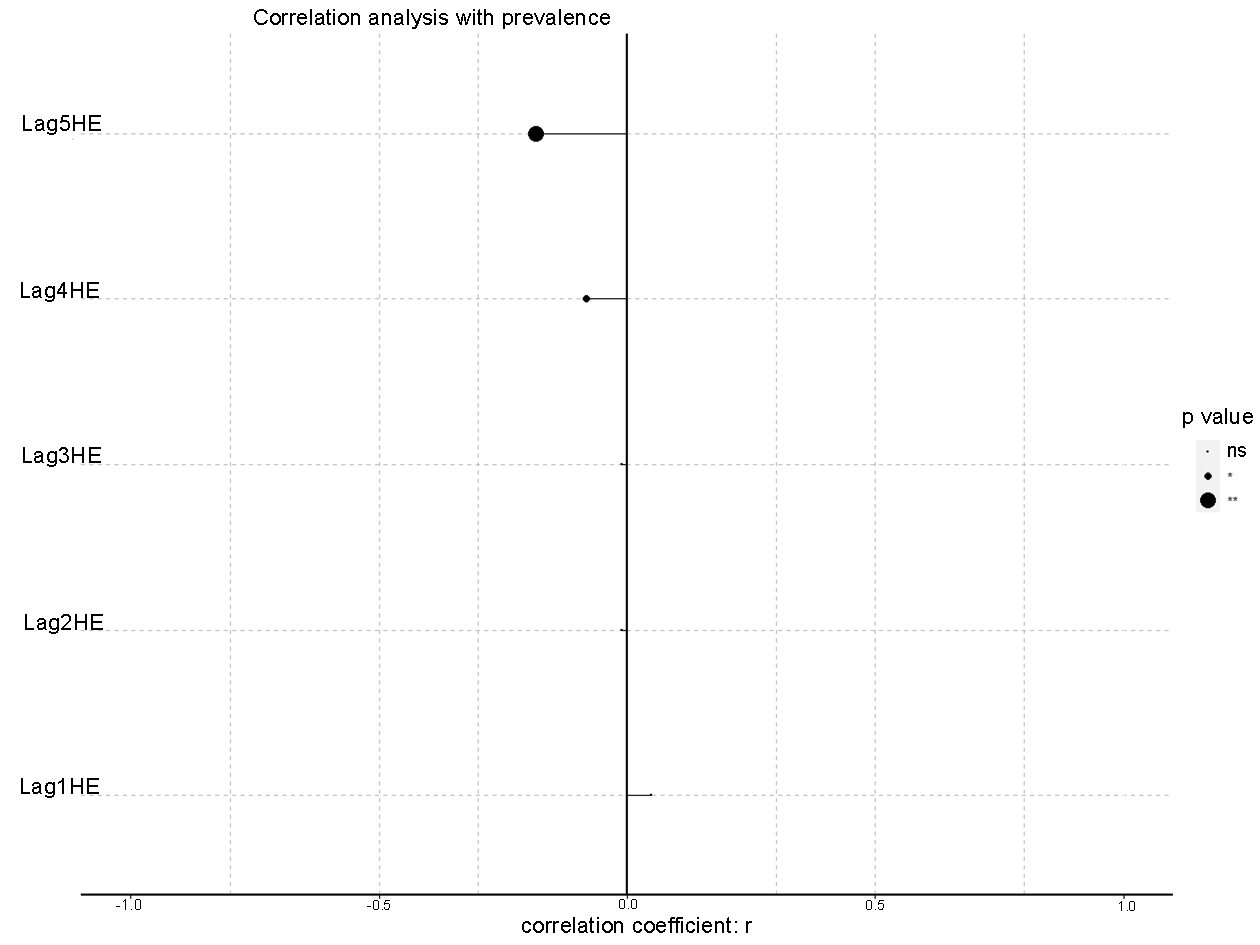


**Figure S3** Visualizing the correlation between health education with a five-year lag and prevalence (Lag1HE (one-year lag in health education), Lag2HE (two-year lag in health education), Lag3HE (three-year lag in health education), Lag4HE (four-year lag in health education), Lag5HE (five-year lag in health education). The figure displays the correlation coefficient on the horizontal axis, indicating both its magnitude and direction. The vertical axis arranges the variables in the order they were inputted, while P-values are denoted by asterisks and dots, <0.01:***, <0.05:**, <0.1:*, ≥0.1:ns.)

**Table S3** The correlation between health education with a five-year lag and disease prevalence

| Variable 1 | Variable 2 | Correlation coefficient | P value |
| --- | --- | --- | --- |
| Prevalence | Lag1HE | 0.05 | 0.54 |
| Prevalence | Lag2HE | -0.01 | 0.90 |
| Prevalence | Lag3HE | -0.01 | 0.90 |
| Prevalence | Lag4HE | -0.08 | 0.06 |
| Prevalence | Lag5HE | -0.18 | 0.02 |


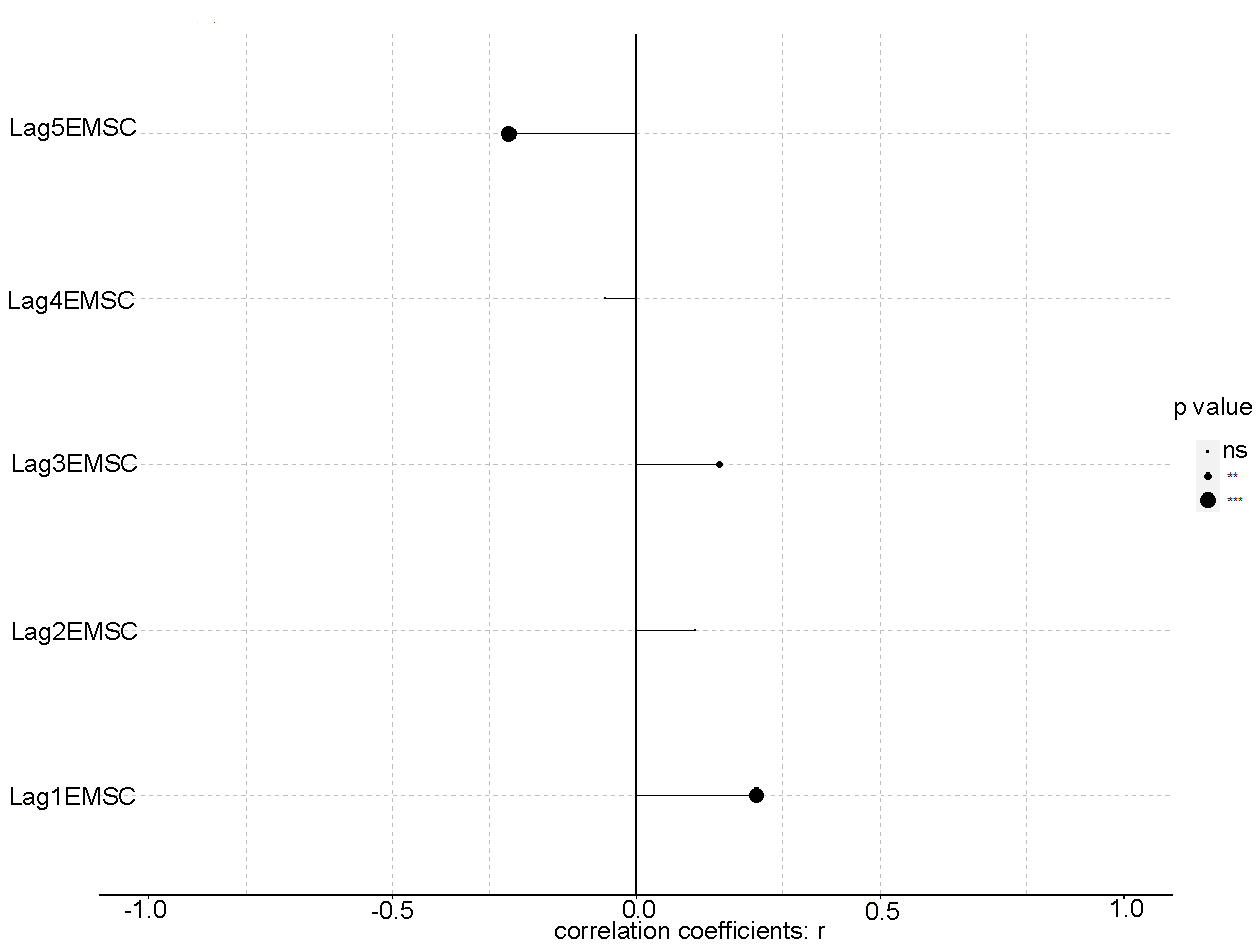


**Figure S4** Visualizing the correlation between environmental modification for snail control with a five-year lag and prevalence (Lag1EMSC (one-year lag in environmental modification for snail control), Lag2EMSC (two-year lag in environmental modification for snail control), Lag3EMSC (three-year lag in environmental modification for snail control), Lag4EMSC (four-year lag in environmental modification for snail control), Lag5EMSC (five-year lag in environmental modification for snail control). The figure displays the correlation coefficient on the horizontal axis, indicating both its magnitude and direction. The vertical axis arranges the variables in the order they were inputted, while P-values are denoted by asterisks and dots, <0.01:***, <0.05:**, <0.1:*, ≥0.1:ns.)

**Table S4** the correlation between environmental modification for snail control with a five-year lag and the prevalence

| Variable 1 | Variable 2 | Correlation coefficient | P value |
| --- | --- | --- | --- |
| Prevalence | Lag1EMSC | 0.25 | 0.00 |
| Prevalence | Lag2EMSC | 0.12 | 0.13 |
| Prevalence | Lag3EMSC | 0.17 | 0.03 |
| Prevalence | Lag4EMSC | -0.06 | 0.44 |
| Prevalence | Lag5EMSC | -0.26 | 0.00 |
