# Supplementary material for: Optimized strategy for schistosomiasis elimination: results from marginal benefit modeling
Source: Parasit Vectors. 2023 Nov 15;16:419. doi: 10.1186/s13071-023-06001-x (PMC10652544; doi:10.1186/s13071-023-06001-x)
Supplement: Supplementary file 5 — Additional file 5: Comparing the prediction accuracy of seven machine learning models. Fig. S5. Visualizing the predictive accuracy of different models. [file 13071_2023_6001_MOESM5_ESM.docx]

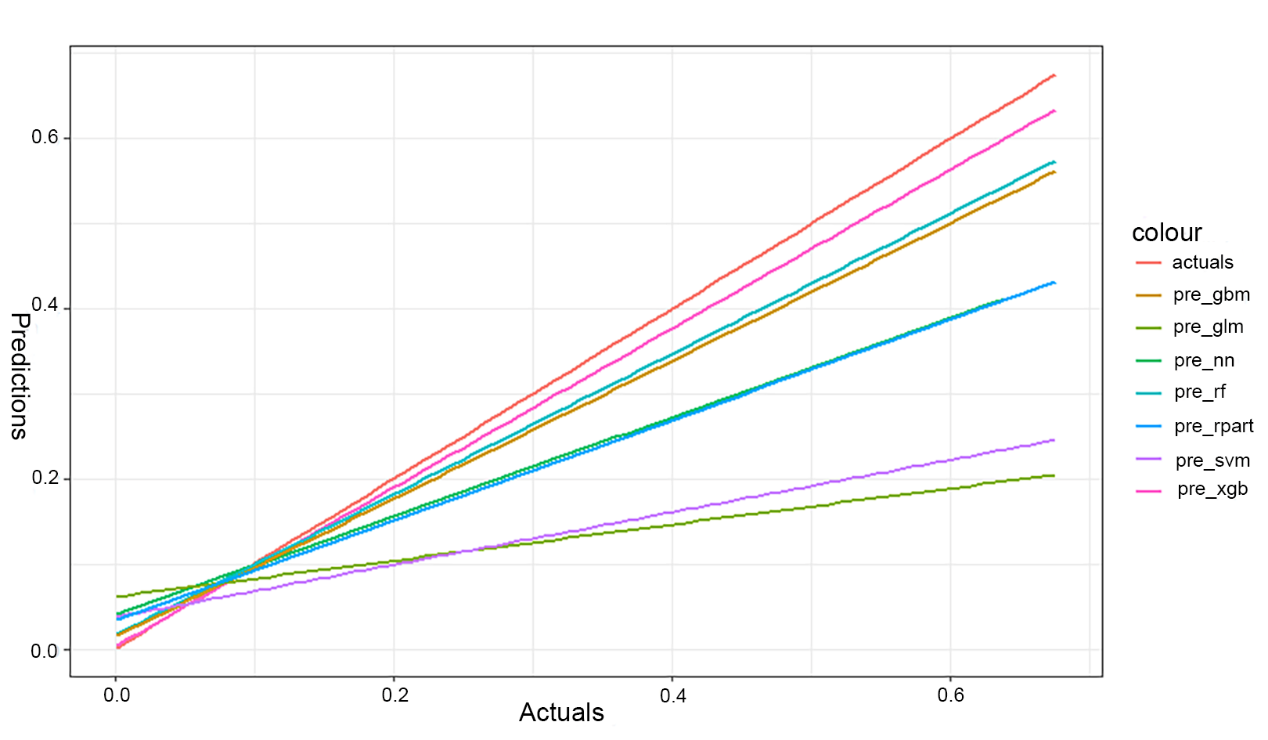


**Fig. S1** Visualizing the predictive accuracy of different models

(The x-axis of Fig. S1 represents the true values, and the y-axis represents the predicted values. The red line represents the performance of the true values in the image, and the closer the model predicted values are to this red line, the higher the prediction accuracy. From the image, it can be seen that the XGBoost model has the highest prediction accuracy)
