# Supplementary material for: Optimized strategy for schistosomiasis elimination: results from marginal benefit modeling
Source: Parasit Vectors. 2023 Nov 15;16:419. doi: 10.1186/s13071-023-06001-x (PMC10652544; doi:10.1186/s13071-023-06001-x)
Supplement: Supplementary file 6 — Additional file 6: Optimal combinations of different size combinations. Fig. S6. Visualization of optimal combinations for different sizes of groups. [file 13071_2023_6001_MOESM6_ESM.docx]

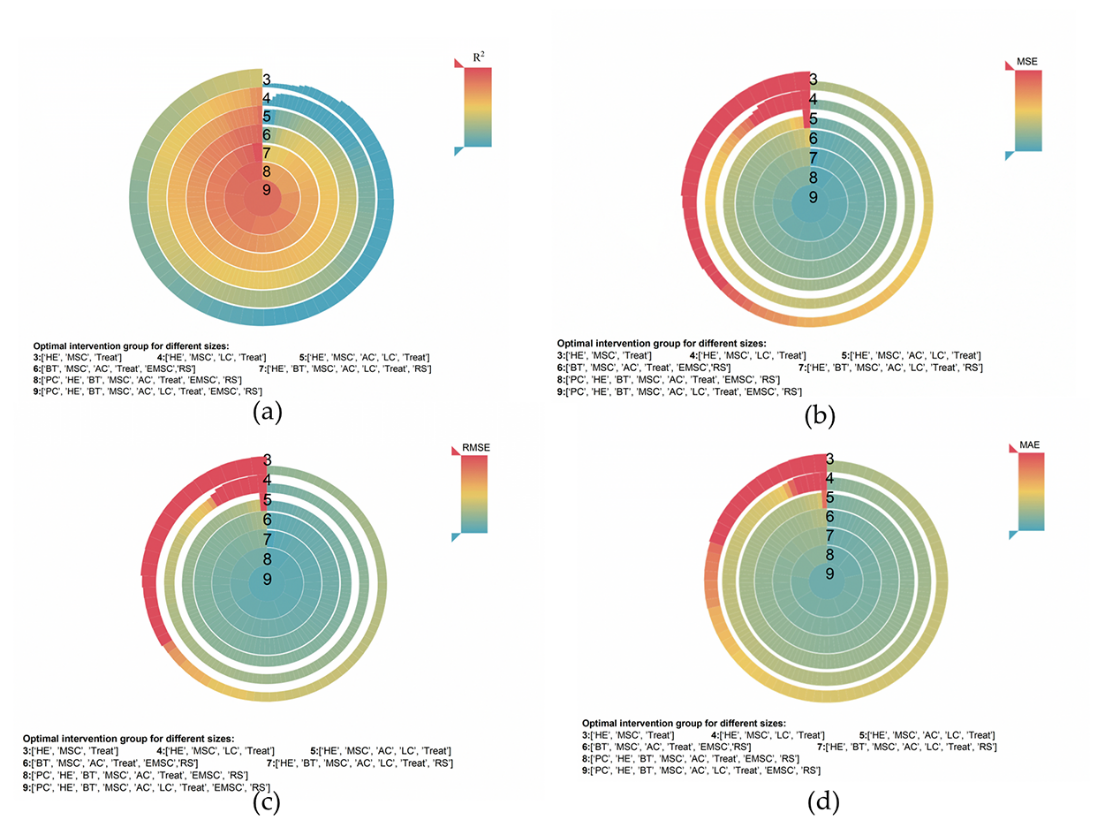


**Fig. S6** Visualization of optimal combinations for different sizes of groups (Comprehensive intervention plans in this study are arranged according to the number of interventions they contain, from the outer ring to the inner ring in Figure 1. The outermost ring represents the combination of three interventions, while the innermost ring represents the combination of nine interventions. Each sector represents a plan, and the color of the sector is related to the R^2^, MSE, RMSE, and MAE of the plan. risk surveillance (RS), molluscicide for snail control (MSC), treatment (Treat), building toilets (BT), animal culling (AC), health education (HE), and livestock chemotherapy (LC). **Fig. S1 (a-d)** separately depicts optimal combination obtained by R^2,^ MSE, RMSE, and MAE in different combinations.)

In addition to the optimal intervention program consisting of seven interventions, from a cost reduction standpoint, it is observed that when the combined intervention includes six or five measures, certain modifications are made relative to the optimal intervention program. In the six-item combination, livestock chemotherapy and health education are excluded, while environmental modification for snail control is added. The five-item combination further excludes risk surveillance. Risk surveillance was removed from the model as the machine learning approach did not incorporate the epidemiological theory of infectious diseases and an increase in prevalence was observed. Due to the promotion of agricultural mechanization, livestock chemotherapy is no longer continued in many endemic areas. Additionally, the lack of effective assessment mechanisms and coverage could be the reason why health education has been replaced by other interventions.
