# Supplementary material for: Optimized strategy for schistosomiasis elimination: results from marginal benefit modeling
Source: Parasit Vectors. 2023 Nov 15;16:419. doi: 10.1186/s13071-023-06001-x (PMC10652544; doi:10.1186/s13071-023-06001-x)
Supplement: Supplementary file 7 — Additional file 7: Priority of the optimal combination. Fig. S7. Contribution ratio of different interventions to prevalence valued by SHAP value in optimal group. [file 13071_2023_6001_MOESM7_ESM.docx]

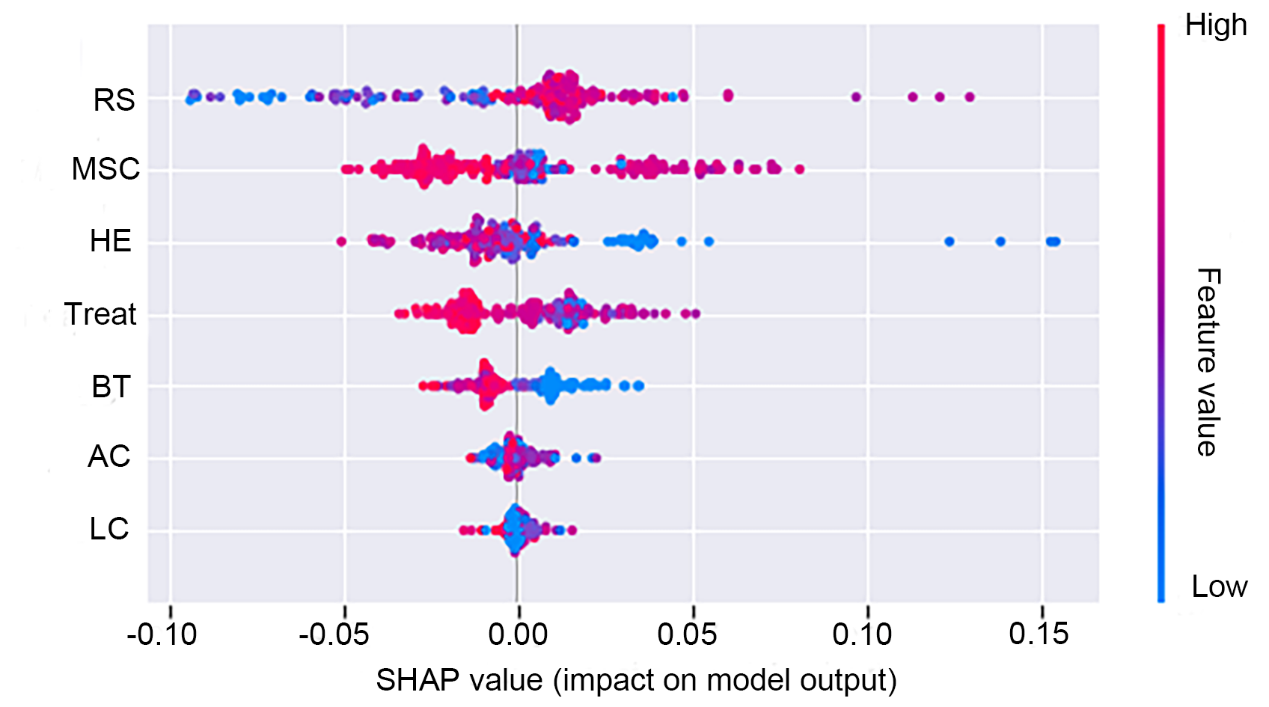


Figure S7 The contribution ratio of different interventions to prevalence valued by SHAP value in optimal group (Risk surveillance (RS), Molluscicide for snail control (MSC), Treatment (Treat), Building toilets (BT), Animal culling (AC), Health education (HE), and Livestock chemotherapy (LC). The vertical axis is sorted by the total sum of SHAP values for each intervention, and the horizontal axis represents the SHAP value (the distribution of the intervention's impact on the model output). Each point represents a sampling county, with overlapping points displayed vertically when their SHAP values are the same, and colors indicate the values of interventions (red corresponds to high values and blue to low values). When most of the red points are distributed on the right side, the feature is positively correlated with disease prevalence, and when they are distributed on the left side, it is negatively correlated. For example, the first row shows that higher RS costs correspond to larger SHAP values, meaning that higher RS costs lead to more cases of schistosomiasis.)

We evaluated the impact of each intervention on the prevalence of schistosomiasis in the optimal program, and the findings are displayed in Figure 1. The cost of risk surveillance was found to be positively correlated with prevalence, while medication for snail control, health education, treatment, building toilets, animal culling, and livestock chemotherapy were negatively correlated with prevalence. The SHAP values showed a decreasing order, reflecting the priority of resource allocation. By eliminating population chemotherapy and environmental modifications for snail control, two resources were made available for allocation to the interventions that were prioritized in the optimized program.
