# Supplementary material for: Optimized strategy for schistosomiasis elimination: results from marginal benefit modeling
Source: Parasit Vectors. 2023 Nov 15;16:419. doi: 10.1186/s13071-023-06001-x (PMC10652544; doi:10.1186/s13071-023-06001-x)
Supplement: Supplementary file 8 — Additional file 8: Optimal combinations of different size combinations in lake and mountainous region. Table S5. Optimal combinations for different sizes of groups in lake and hill type endemic area. [file 13071_2023_6001_MOESM8_ESM.docx]

**Table S5** Optimal combinations for different sizes of groups in lake and hill type endemic area

| Number of interventions included in combination | Label | Cost | R^2^ | MSE | RMSE | MAE |
| --- | --- | --- | --- | --- | --- | --- |
| Lake | | | | | | |
| 3 | ['HE', 'MSC', 'RS'] | 4.2502 | 0.99973 | 0.000001877 | 0.00137 | 0.000963119 |
| 4 | ['AC', 'HE', 'Treat', 'RS'] | 4.8471 | 0.999849 | 0.000001051 | 0.001025 | 0.000738731 |
| 5 | ['HE', 'MSC', 'LC', 'Treat', 'RS'] | 6.4799 | 0.999881 | 0.000000827 | 0.000909 | 0.000646129 |
| 6 | ['HE', 'BT', 'LC', 'PC', 'Treat', 'RS'] | 6.1056 | 0.999889 | 0.000000773 | 0.000879 | 0.00061141 |
| 7 | ['AC', 'HE', 'BT', 'MSC', 'LC', 'Treat', 'RS'] | 8.7433 | 0.999912 | 0.000000615 | 0.000784 | 0.00058682 |
| 8 | ['AC', 'HE', 'BT', 'LC', 'PC', 'Treat', 'EMSC', 'RS'] | 8.1592 | 0.999892 | 0.000000751 | 0.000866 | 0.000603018 |
| 9 | ['AC', 'HE', 'BT', 'MSC', 'LC', 'PC', 'Treat', 'EMSC', 'RS'] | 10.13344 | 0.999885 | 0.000000802 | 0.000896 | 0.000610021 |
| Hill | | | | | | |
| 3 | ['HE', 'Treat', 'RS'] | 3.990633 | 0.99993 | 0.000000649 | 0.000805 | 0.000616 |
| 4 | ['MSC', 'LC', 'Treat', 'RS'] | 5.703409 | 0.999944 | 0.00000052 | 0.000721 | 0.000559 |
| 5 | ['HE', 'MSC', 'LC', 'Treat', 'RS'] | 6.406544 | 0.999954 | 0.000000432 | 0.000657 | 0.000451 |
| 6 | ['HE', 'MSC', 'LC', 'PC', 'Treat', 'RS'] | 7.342574 | 0.999958 | 0.000000395 | 0.000628 | 0.000427 |
| 7 | ['AC', 'HE', 'BT', 'MSC', 'LC', 'EMSC', 'RS'] | 7.558849 | 0.999961 | 0.000000364 | 0.000603 | 0.000448 |
| 8 | ['AC', 'HE', 'BT', 'MSC', 'LC', 'PC', 'Treat', 'RS'] | 9.41643 | 0.999963 | 0.000000346 | 0.000588 | 0.000421 |
| 9 | ['AC', 'HE', 'BT', 'MSC', 'LC', 'PC', 'Treat', 'EMSC', 'RS'] | 10.11815 | 0.999962 | 0.000000356 | 0.000597 | 0.000427 |

As shown in Table 1, in both types of endemic areas, the prevention and control effect obtained gradually approached the overall value as the number of interventions and the cost of interventions increased. The optimal intervention combination in the hill-type region consists of 8 interventions: animal culling, health education, building toilets, snail control molluscicide, population and livestock chemotherapy, treatment, and risk surveillance. The second-best combination excludes livestock chemotherapy and building toilets from the optimal combination. In the lake region, the optimal combination includes 7 interventions, with the second-best combinations consisting of 6 or 5 interventions still being relatively effective, and with minor differences from the optimal combination of 7 interventions. The optimal intervention combination in the lake-type region consists of 8 interventions: animal culling, health education, building toilets, snail control molluscicide, livestock chemotherapy, treatment, and risk surveillance. The second-best combination excludes animal culling and building toilets from the optimal combination. Although using all 9 interventions is effective, it is not the most optimal approach, potentially due to resource constraints.
